# Transgender Athletes in Sports Competitions: How Policy Measures Can Be More Inclusive and Fairer to All

**DOI:** 10.3389/fspor.2021.704178

**Published:** 2021-07-14

**Authors:** Allie Reynolds, Alireza Hamidian Jahromi

**Affiliations:** ^1^Princeton University, Princeton, NJ, United States; ^2^Plastic and Reconstructive Surgery Department, Rush University Medical Center, Chicago, IL, United States

**Keywords:** transgender athletes, athletics, sports policy, inclusivity, fairness

## Introduction

Recently, there has been much debate over the inclusion of transgender athletes in elite athletic competitions. Since the transgender population in the United States and worldwide is increasing every year (Meerwijk and Sevelius, 2017) and with it the transgender athletic population, it's important to establish athletic policies that are both inclusive and fair to avoid future conflicts. In this article, environmental and social barriers to transgender athlete participants as well as biological differences related to athletic performance are examined. A review of the current athletic policies and suggestions for potential policy updates are provided. We acknowledge that this is a relatively under-researched field and that there is no clear-cut solution. However, we believe this topic is important and we hope to contribute to the ongoing discussion.

## Barriers to Participation

It's important to first address some of the barriers transgender athletes face to participate in sports competitions before examining the current policies behind their participation. A recent study showed that transgender men (TGM) are significantly more likely to participate in team sports than transgender women (TGW) but that this difference is not apparent for individual sports (López-Cañada et al., 2020). Transgender women have previously stated that the primary barrier to their participation is the lack of an environment that is both inclusive and comfortable (Jones et al., 2017), and this could contribute to their decreased participation in team sports. More specifically, TGW perceive their voices to be a contributing barrier in their lack of participation (Stewart et al., 2020). Sports that are strongly gendered create an environment for these athletes that makes them feel anxious that speaking out or cheering for their teammates could result in them not being identified as women (Stewart et al., 2020). Similarly, sports clothing may serve as a barrier to participation because it can be physically revealing. For example, a TGW who has not had bottom surgery might use a “tucking” technique to hide the bulge of the penis and testicles. Sports bras can relatedly impact transgender athletes: TGW might add padding to their bras and TGM might bind their chests. Any of these actions could be uncomfortable to the athlete and/or hinder performance in sports competitions. Additionally, locker rooms and other team spaces are often strongly segregated by gender and transgender athletes may be excluded from areas that match their gender identity. Restricting athletes from such areas, regardless of whether they are allowed to participate, may have the effect of causing athletes feeling separated from their teammates and their gender identity invalidated (Cunningham et al., 2018).

There is certainly an additive effect of the numerous barriers to participation discussed, but one of the most important and in some cases least understood barriers is stigma. Although stigma is not a novel concept, stigma in how it impacts transgender athletes is more of a recent phenomenon. The numerous roles of stigma are often under-recognized (Hatzenbuehler, 2017), and acknowledging it prior to developing new policies could help to combat some of its negative effects. Transgender stigma, in general, limits opportunities and can have extremely negative effects on mental and physical health (Hughto et al., 2015). Stigma acts at numerous levels (e.g., structural, interpersonal, and individual), and adopting interventions to address and combat the negative effects of stigma at all of these levels is an important aspect of developing any new policy (Hughto et al., 2015), especially when this policy aims to include transgender athletes. This is especially significant in developing sports policy that addresses youth athletes, as transgender stigma can be heightened when geared toward transgender youth and adolescence is a critical point to target interventions (Hatzenbuehler and Pachankis, 2016; Hatzenbuehler, 2017).

To add to these social and environmental barriers, athletic policy restrictions have also contributed to a decrease in participation of the transgender individuals in competitive sports (Jones et al., 2017). The lack of consensus among the various athletic governing bodies makes it even more difficult to determine the exact policies to include transgender athletes in sports competitions. Acknowledging these barriers to participation is an extra element that should be included in the adoption of new athletic policies regarding transgender athletes.

## Biological Differences Related to Athletic Performance

The current debate over including transgender athletes in sports competitions (in their current state) is centered on biological differences, most notably those between transgender and cisgender women. Performance disparities based on “assigned sex at birth” vary across sports—they are known to be the lowest for swimming and highest for track and field events (Bassett et al., 2020). These differences in athletic performance don't appear until after puberty and are thought to be most likely due to increased circulating testosterone levels in the “male” assigned sex at birth athletes when compared to the “female” assigned sex at birth athletes (Handelsman et al., 2018). However, there is a general lack of data showing that higher testosterone levels are correlated with improved athletic performance (Karkazis, 2019).

Despite the lack of evidence, hormone therapies are currently being employed by TGW to suppress their testosterone levels to those more similar to cisgender women to comply with competition regulations. Interestingly, the muscular advantage of TGW over cis-gender women is only minimally reduced after testosterone suppression (Hilton and Lundberg, 2021). This suggests that in certain athletic competitions which rely on muscles mass and explosive strength, TGW will still have a physical advantage even if they are able to lower their testosterone levels to the officially requested threshold. Other hormone therapies have been successful at decreasing hemoglobin levels in TGW after only 4 months, but remain unsuccessful at decreasing strength, lean body mass, and muscle area even if undergone for 36 months (Harper et al., 2021). Although only slight changes are seen in TGW after hormone therapies, this is not the case for TGM. After only 1 year of gender-affirming hormone treatment, TGM were able to significantly increase muscle mass and strength (Wilk et al., 2020). Without a scientific evidence that testosterone levels are mainly responsible for athletic performance discrepancies between transgender and cisgender women, TGW could be undergoing unnecessary treatments. More research is needed to show this link before athletic governing bodies can enforce decreased testosterone policies as a requirement for TGW to attend competitions.

While proposed methods for categorization may be considered as a “commonsense and clear-cut assessment” by many, they have all failed as they were not scientifically driven (Karkazis, 2019). Authorities have used physical examination in 1960's, chromosomal testing in 1970's, and testosterone measurements in 2010's and 2020's for “sex testing” athletes to allow them to participate in competitions (Karkazis, 2019). “Physical examination of genitals,” “chromosomes,” “gonads,” and more recently “hormones” have all been used in “sex testing” and as evidence for categorization in sports through the history albeit without success; mostly due to the fact that they were not scientifically based and only considered “common sense.”

## Current Athletic Policies

A fundamental issue regarding the current sports policies on transgender athletes is that the governing bodies of different athletic organizations have very different policies these athletes must follow to be included in sports competitions. In 2019, the International Olympic Committee (IOC) restricted all athletes from competing in the female category unless they lowered their natural testosterone levels below 5 nmol/L (Harper et al., 2018a). This level was recently increased in 2021 to 10 nmol/L and the additional requirement of these levels being maintained for at least 12 months prior to competition was added (Hilton and Lundberg, 2021). Unlike the IOC, the National Collegiate Athletic Association (NCAA) has much less explicit guidelines for the inclusion of transgender athletes. They only require that TGW must complete at least one year of hormonal suppressive therapy to participate on a women's team, but do not require natural testosterone levels to be below a specific level (NCAA Office of Inclusion, 2011). The NCAA's policies have not been updated since 2011, suggesting that there could be room for improvement based on new and more updated research.

As touched upon previously, the center of the debate over the inclusion of transgender athletes in sports competitions is the physical advantages that TGW could have. However, the “female” category for sports in general is ambiguous and not established in the same manner universally (Ingram and Thomas, 2019). In order for competitions to remain fair, universal rules need to be created regarding the inclusion or exclusion of transgender athletes. Currently, policies and perceived fairness of inclusion vary immensely at the level of sporting competition (Tanimoto and Miwa, 2021), meaning that there is a large difference in how transgender athletes are perceived at professional and non-professional levels. Setting the standards for their inclusion at the professional level, may result in other levels of sporting competitions (e.g., recreation leagues, high school athletics, sports clubs, etc.) following suit. However, it has also been argued that the aim of athletics at these non-professional levels is mass participation, and therefore more restrictive guidelines should be avoided (Cunningham et al., 2018; Buzuvis, 2019, 2021; Tanimoto and Miwa, 2021).

Both the medical and the scientific communities need to provide input to help guide the creation of such rules (Ingram and Thomas, 2019), especially with hormone therapy expansions as well as increased research into the link between testosterone levels and increased athletic performance. While physicians will play an influential role in developing new sports policy, it is important to also acknowledge the roles of sports managers and others who have experience in sport governance and development. Opening conversations among all of these individuals is the first step to ensuring the success and implementation of new policies at all levels of sporting competitions.

## Proposed Solutions

Numerous solutions have been proposed to include transgender athletes in sports competitions while being fair to all athletes. Since numerous nations around the world already allow a “third legal gender,” some have proposed extending this idea to elite sports as a separate category for athletes who identify as this gender (Harper et al., 2018b). A problem with this idea that it still excludes athletes who don't identify as the third legal gender, leaving some athletes without a category in which they can compete. Others suggest employing an algorithm that includes all athletes and divides them into categories based on both physiological and social parameters (Anderson et al., 2019). This idea is still relatively new, and more research is needed to determine how inclusive this approach is and how effective it would be to enact.

Others suggest reforming sports policies to favor participation based on gender identity and not on biological sex (Buzuvis, 2019, 2021). This solution argues that in general, U.S. policies are on the side of inclusion and that this can readily extend into athletic policies, especially for youth athletes (Buzuvis, 2019, 2021). While there are certainly merits to this argument in terms of inclusion, it is difficult to completely ignore the biological arguments discussed previously. Thus, a solution that balances both inclusivity and fairness is the best approach to this problem in particular.

The most important parameters when assessing methods to improve current sports policies are determining how inclusive a policy is to transgender athletes and how fair it is to all athletes involved in competitions. Many suggest adding more categories under which athletes can compete (Knox et al., 2019), upholding inclusivity without compromising fairness. However, it is unclear how many categories would need to be added to accomplish this feat and if athletic organizations can financially support a large number of athletic categories competing under each sport. For this reason, we suggest adding a third category to elite sports similar to that suggested above, but without the legal third gender requirement. This category would be considered “open,” meaning that any athlete can compete regardless of their gender identity. Male and female sports categories would still be included in this idea but adding an “open” category is more inclusive to all athletes who wish to participate. As we believe that gender is a no longer a binary concept, having an open category supports the inclusion of non-binary, transgender, and genderqueer groups of individuals in sports competitions. While this idea has its advantages and disadvantages, we believe that the language used in naming a third category is especially important and the term “open” is more inclusive than previous suggestions.

## Discussion

The population in the United States, similar to the rest of the world, is constantly changing and it's imperative that elite athletics mirrors these changes. This is especially relevant for the community of transgender athletes as they should be included in sports competitions in a fair and inclusive manner. It is clear that more research is needed to determine what advantages transgender athletes, particularly TGW, could have in athletic competitions. This needs to be accomplished prior to making definitive policy statements regarding the inclusion or exclusion of transgender athletes (Johnston, 2020). In the meantime, current policies need to be careful in the language used in order to promote inclusivity.

## Author Contributions

AH conceptualized the paper and AR wrote the first edition of the manuscript. AR and AH contributed to the manuscript with their expertise, read, edited, and approved the submitted version. Both authors contributed to the article and approved the submitted version.

## Conflict of Interest

The authors declare that the research was conducted in the absence of any commercial or financial relationships that could be construed as a potential conflict of interest.
